# Electron-Phonon Coupling and its implication for the superconducting topological insulators

**DOI:** 10.1038/srep08964

**Published:** 2015-03-10

**Authors:** Xiao-Long Zhang, Wu-Ming Liu

**Affiliations:** 1 Beijing National Laboratory for Condensed Matter Physics, Institute of Physics, Chinese Academy of Sciences, Beijing 100190, China

## Abstract

The recent observation of superconductivity in doped topological insulators has sparked a flurry of interest due to the prospect of realizing the long-sought topological superconductors. Yet the understanding of underlying pairing mechanism in these systems is far from complete. Here we investigate this problem by providing robust first-principles calculations of the role of electron-phonon coupling for the superconducting pairing in the prime candidate Cu*_x_*Bi_2_Se_3_. Our results show that electron-phonon scattering process in this system is dominated by zone center and boundary optical modes, with coexistence of phonon stiffening and softening. While the calculated electron-phonon coupling constant *λ* suggests that *T_c_* from electron-phonon coupling is 2 orders smaller than the ones reported on bulk inhomogeneous samples, suggesting that superconductivity may not come from pure electron-phonon coupling. We discuss the possible enhancement of superconducting transition temperature by local inhomogeneity introduced by doping.

The successful fabrication of topological insulators (TIs)^1^,^2^,^3^,^4^ has stimulated the search for other exotic topological matters. Among them the topological superconductors (TSCs)^2^ are of particular interest owing to its topologically protected gapless surface states consisting of massless Majorana fermions, which may find potential application in future topological quantum computation. The close relationship between TIs and TSCs^2^,^5^,^6^ indicates that topological superconductivity may born from TI. Indeed, there have been several attempts to induce topological superconductivity in TI^7^,^8^,^9^,^10^,^11^,^12^. In particular, the recent observation of superconductivity in doped topological insulators^13^,^14^,^15^,^16^ has received great attention and has been considered as prime candidates for TSCs.

The superconductivity in these doped topological insulators is unique since it occurs at relative low carrier concentration, and particularly the Dirac surface states remain intact with the onset of superconductivity in the case of Cu*_x_*Bi_2_Se_3_^14^. Shortly after the experimental discovery, Fu *et al.*^17^ argued that Cu*_x_*Bi_2_Se_3_ favors spin-triplet paring with odd-parity owing to its strong spin-orbit coupled band structure, and may realize a time-reversal-invariant (TRI) TSC. Although there is controversy on whether the Fermi surface of superconducting Cu*_x_*Bi_2_Se_3_ encloses odd number of TRI momenta^18^,^19^, which is key to a true TSC^17^, the prospect for a topologically nontrivial paring in this system is quite appealing. Indeed, subsequent point-contact spectroscopy measurements^15^ seem observe the zero bias conductivity peak which signifies the presence of Majorana surface states. But the result was challenged by other tunneling spectra measurements^20^,^21^, leaving the topological aspect of superconductivity in this system has yet to be confirmed.

Despite much work concerning these systems to date^22^,^23^,^24^,^25^,^26^,^27^,^28^, the microscopic paring mechanism of the superconducting topological insulators remains elusive. On the one hand, unconventional superconductivity is generally associated with systems with strong electron correlations which seems unlikely for the typical topological insulators with *sp* electrons like Bi_2_Se_3_. On the other hand, whether electron-phonon (*e-ph*) coupling, which plays a central role in conventional BCS superconductivity, would realize the required unconventional paring is still an open question^17^,^24^,^25^. To address this problem, a comprehensive understanding of phonons and *e-ph* coupling in doped topological insulators is thus strongly called for.

In this Report, we present a systematic study of phonons and role of *e-ph* coupling for the representative system Cu*_x_*Bi_2_Se_3_ for a range of doping with accurate first-principles calculations. The *e-ph* properties are evaluated robustly by sampling the Brillouin zone (BZ) with several tens of thousands of *inequivalent* electron and phonon momenta. Our results reveal interesting renormalization of phonon dynamics due to electron doping, with coexistence of optical phonon stiffening and softening at zone center and boundary, signifying that the whole *e-ph* scattering process is dominated by these modes. Nevertheless, the obtained *e-ph* coupling constant *λ* is 0.28 for the optimally doped (*x* = 0.12) Bi_2_Se_3_, resulting in a maximum *T_c_* of only ~0.03 K, which is significantly smaller than the ones reported on inhomogeneous bulk^13^,^14^,^15^,^16^ but in line with very recent experiment where disorder and inhomogeneity are strongly suppressed^29^. Our results thus indicate that the superconductivity in Cu*_x_*Bi_2_Se_3_ may not be explained by only resorting to *e-ph* mechanism. We discuss the possibility of enhancement of superconducting transition temperature by local inhomogeneity introduced by copper doping.

## Results

### Doping induced strong renormalization of phonons dynamics

**Figure 1:**
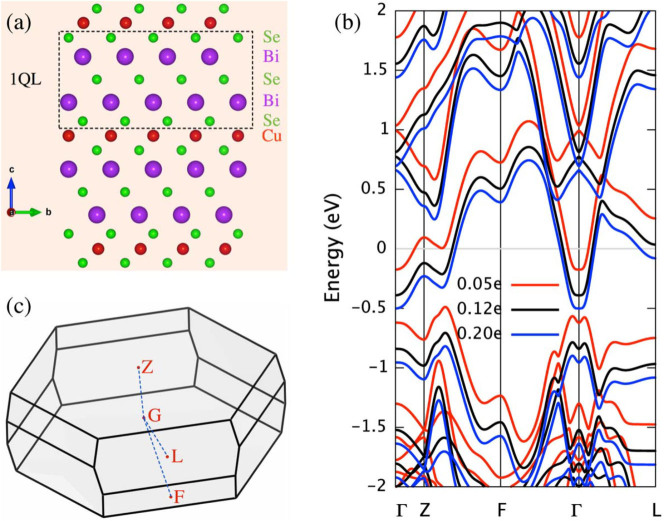
The crystal structure of Cu*_x_*Bi_2_Se_3_ and energy band evolution with electron doping. The crystal structure (a) and Brillouin zone (c) of Cu*_x_*Bi_2_Se_3_. (b) the electronic energy bands for 3 representative Cu doping (*x* = 0.05*e* for an ellipsoidal Fermi surface, *x* = 0.12*e* for a nearly cylindrical Fermi surface and *x* = 0.2*e* corresponding to even more complex Fermi surface topology). To highlight the rigid shift of whole energy bands with electron doping, the Fermi level of each case has been set to 0.

The Bi_2_Se_3_ crystallize in a rhombohedral structure with stacked Se-Bi-Se-Bi-Se quintuple layer (QL) as building blocks. Experimental data have consistently shown that copper atoms are mainly interpolated into the van der Waals gap between quintuple layers of Bi_2_Se_3_ and act as electron donor^13^,^14^,^30^. And the bulk electronic structures are virtually intact upon copper doping apart from a rigid shift of whole energy bands^30^,^31^ (see Fig. 1(b)). Hence we simulate doped topological insulator Bi_2_Se_3_ with the rigid-band approximation, where the excess of electron is compensated by uniform background of positive charge.

The vibrational properties of nominally undoped bulk Bi_2_Se_3_ have been extensively studied using Raman spectroscopy^32^,^33^ and all 4 Raman active optical phonon have been observed^33^. On the other hand, most available phonon data from fully-relativistic first-principles calculations are, however, unsatisfactory due to the presence of dynamical instability^34^,^35^. Our phonon dispersion does not suffer from this problem and the calculated zone center optical modes, which can be classified according to the irreducible representation of the point group of bulk Bi_2_Se_3_ as Γ = 2(*E_g_* + *A*_1*g*_ + *E_u_* + *A*_2*u*_), agree well with experimental one^33^. The phonon spectrum extends up to 21.8 *meV*, and the vibrations of Se atoms mainly account for the top 9 optical branches and well separated in energy from Bi due to large mass difference.

**Figure 2:**
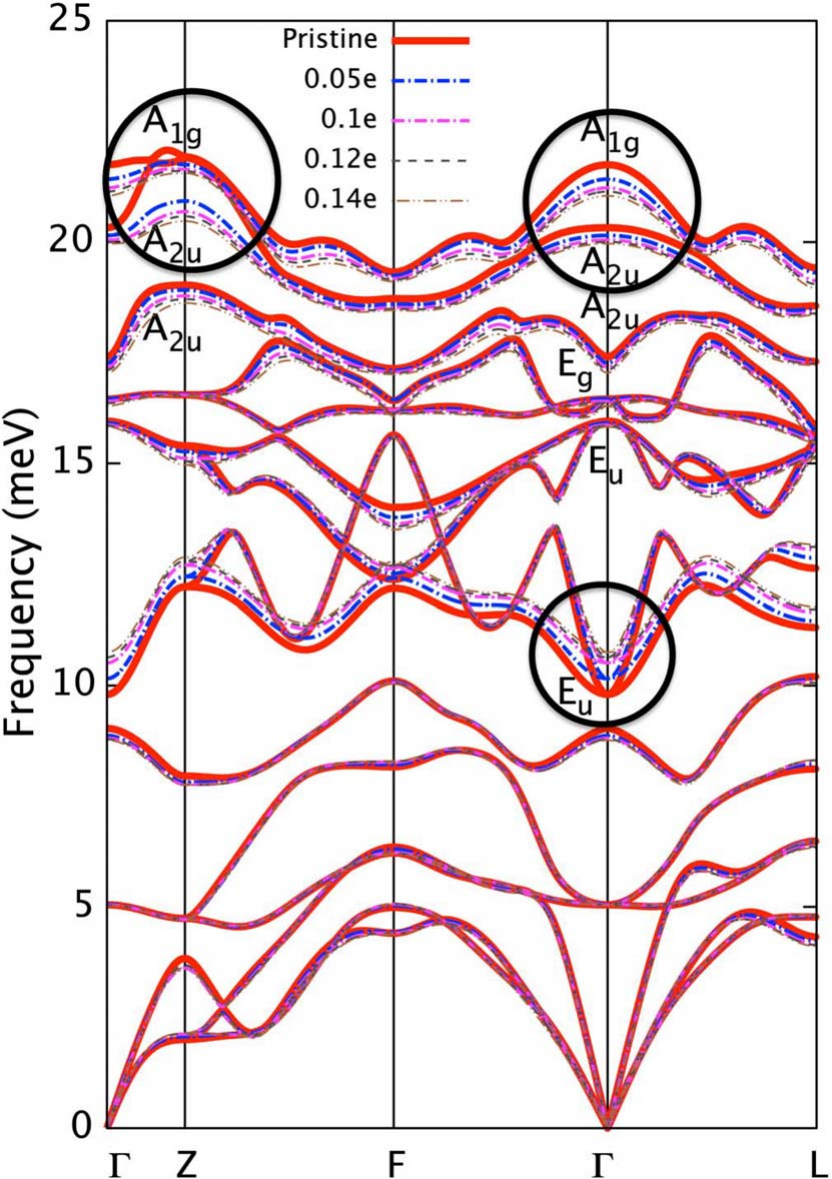
The electron doping dependence of phonon dispersion. Phonon modes of interest are labeled according to the irreducible representation of point group of bulk Bi_2_Se_3_. The black circles highlight the major modifications of phonon modes (*A*_2*u*_ at Z; *A*_1*g*_, *A*_2*u*_ and *E_u_* at Γ) with copper doping.

When doped with copper atoms, the bulk conduction band of parent compound Bi_2_Se_3_ is partially filled due to charge transfer, and eventually develops superconductivity with copper concentration exceeds 0.10 when cooled down. Fig. 2 shows the phonon dispersions of bulk Cu*_x_*Bi_2_Se_3_ corresponding to several doping levels (0 ≤ x ≤ 0.14) from a fully-relativistic calculation. As can be seen, while leaving the electronic structure almost unchanged^30^, electron doping strongly alters the vibrational properties of doped system, especially as reflected in the pronounced softening of the highest *A*_2*u*_ zone boundary modes (Z point) and the coexistence of modes stiffening (*E_u_*) and softening (*A*_1*g*_ and *A*_2*u*_) around zone center (Γ point). This doping-dependent phonon renormalization is most effect when doping starts (*x* = 0.05) and quickly saturates as the doping continues (especially at Z point as can be seen from Fig. 2), reflecting different phonon dynamics of bulk insulating Bi_2_Se_3_ and electron-doped one due to electron screening. We note that in most experiments, the bulk sample of Bi_2_Se_3_ is usually slightly electron-doped owing to the presence of Se vacancies. Hence the abrupt change of phonon frequencies (Z point) may not be easily observed. Indeed, previous Raman spectroscopy measurement^36^ showed no sign of significant change of phonon frequencies around zone center with Cu doping. Nevertheless, the special phonon stiffening and softening with electron doping here signifies these modes would dominate the *e-ph* scattering process. Actually, according to previous first-principles calculations^3^, the energy bands around Fermi level are dominated by the hybridized *p_z_* orbitals from outermost Se atoms in a unit cell. And the *A*_2*u*_ mode at Z and Γ involves out-of-phase motions of Bi and Se atoms along the z-direction. Hence with electron doping, the partially occupied anti-bonding states would strongly interact with this phonon pattern, resulting in relatively large phonon linewidths as will be shown below.

### Phonon linewidths from electron-phonon couping

**Figure 3:**
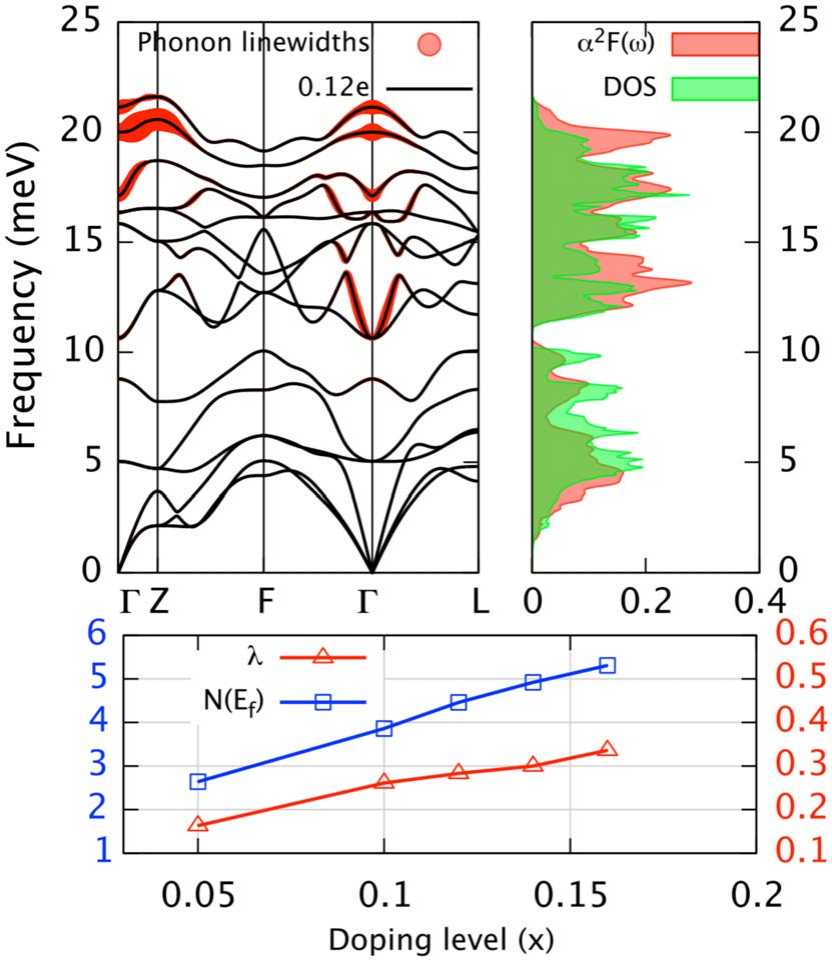
The phonon linewidths for optimally doped (*x* = 0.12*e*) Cu*_x_*Bi_2_Se_3_ and doping dependence of electron-phonon properties. Upper left: The radius of red circle is proportional to the magnitude of phonon linewidths. Upper right: Comparison of Eliashberg spectral function and phonon density of states for optimally doped case (*x* = 0.12*e*). Lower: The doping dependence of electron-phonon coupling constant *λ* (red point line) and electronic density of states at Fermi level *N*(*E_f_*) (blue point line).

The electron doping induced stiffening and softening of specific phonon modes is a clear sign of moderate *e-ph* interaction in this system. This is manifested in the relatively large phonon linewidths around zone center and zone boundary for the optimally doped (*x* = 0.12) Cu*_x_*Bi_2_Se_3_ (see Fig. 3). In calculating phonon linewidths, we have sampled the BZ with 50 × 50 × 50 *inequivalent* electron wave vectors and the *δ* in Eq. (4) is replaced with 0.001 Ryd. From Fig. 3 we can see the significant phonon linewidths mainly lie in the top 9 optical branches which involve in-plane Se phonons and restricted to certain modes around the zone center (Γ) and zone boundary (*Z*) where phonon stiffening and softening occur. The phonon linewidths at other regions where phonon momentum *q* > 2*k_f_* (*k_f_* is the electronic Fermi momentum) are negligible. This localized distribution of phonon linewidths resembles that in MgB_2_^37^,^38^, where the *E*_2*g*_ in-plane phonons near zone center strongly couples with partially occupied *σ*-bonding states and results in the largest *e-ph* coupling. This may suggest that Cu*_x_*Bi_2_Se_3_ is a good *e-ph* superconductor, but this is unfortunately not the case as will be discussed later.

There is another question need to be answered, namely how phonon linewidths evolve with electron doping, since the topology of Fermi surface (FS) of Cu*_x_*Bi_2_Se_3_ undergoes significant change from being an 3-D ellipsoid to 2-D cylindrical one^19^,^39^. It turns out that the doping dependence of phonon linewidths is negligible although the scattering phase space is increasing monotonically.

### Role of *e-ph* couping for superconductivity

Now let us turn to the *e-ph* coupling contribution to the superconductivity in Cu*_x_*Bi_2_Se_3_. It has been shown the superconductivity of Cu*_x_*Bi_2_Se_3_ occurs for a wide range of doping level (0.10 < *x* < 0.60)^40^, and the highest superconducting transition temperature of 3.8 K is achieved at *x* = 0.12. Within the framework of Migdal-Eliashberg theory^41^,^42^, the superconductivity arising from *e-ph* coupling is characterized by several quantities^43^,^44^, the most important ones are Eliashberg spectral function
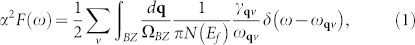
and the dimensionless *e-ph* coupling constant 
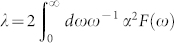
. As mentioned above, a reliable *λ* needs dense sampling of both phonon and electron momentum. This is achieved here with the aid of Wannier interpolation, where the electronic and phonon states as well as *e-ph* coupling matrix elements are calculated on a fine mesh with 36 × 36 × 36 and 30 × 30 × 30 *inequivalent* electron and phonon momenta, respectively. This dense grid ensures the convergence of *λ* to within 0.005.

**Figure 4:**
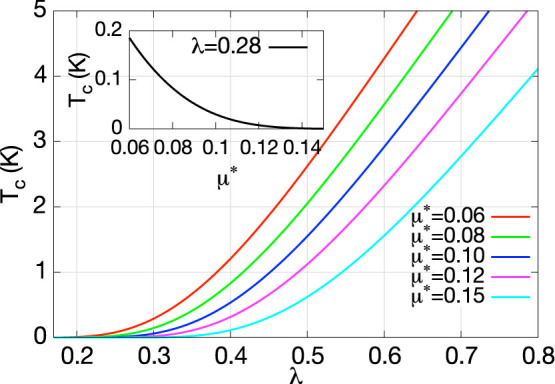
The dependence of *T_c_* on *λ* and μ*. *T_c_* has been plotted against *λ* and μ*, from which we can estimate the lower bound of *λ* to be ~0.6 to match the experimental *T_c_* of 4 K. The inset indicates the calculated *T_c_* is far less than the experimental one for optimally doped case.

Fig. 3 summarizes our *e-ph* results for Cu*_x_*Bi_2_Se_3_. From the comparison of phonon DOS and Eliashberg spectral function we find that the *α*^2^*F*(*ω*) generally follow the trend of phonon DOS for the low-lying Bi modes (*ω* < 10 *meV*) while deviate for the high-lying Se modes, and the discrepancy is significant around 13 *meV* and 20 *meV*. Such behavior has been seen in MgB_2_, where the most significant contribution to the remarkable high *T_c_* of 39 K comes from phonon modes around 60 *meV*^38^. Despite this resemblance, the *e-ph* coupling constant *λ* for the optimally doped (*x* = 0.12) Cu*_x_*Bi_2_Se_3_ is 0.28. The corresponding transition temperature *T_c_* can be obtained from Allen-Dynes formula^43^:
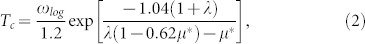
where
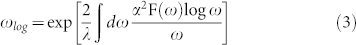
is the logarithmically averaged phonon frequency. From Fig. 4 we can conclude that for typical retarded Coulomb repulsion μ* of 0.10, the estimated *T_c_* is only 0.03 K, which is 2 orders smaller than experimental value. If we assume a constant *ω_log_* (~11 *meV*), to match the experimental value of *T_c_* the required *λ* would be 0.6–0.7 (with reasonable Coloumb μ* of 0.06–0.12, see Fig. 4), which is ~2 times larger than present one. Since the electronic DOS at Fermi level *N*(*E_f_*) is 4.46 states/Ryd/spin/(unit cell) which is almost identical to that in MgB_2_, it is clear that the major obstacle prevents Cu*_x_*Bi_2_Se_3_ from being a good *e-ph* superconductor as MgB_2_ (with *λ* ~ 1) is the lower characteristic phonon frequency (~60 *meV*^37^ for MgB_2_ but ~11 *meV* here) or Debye temperature (~900 K^45^ for MgB_2_ but ~180 K^46^ here).

In Fig. 3 we also plot the electron doping dependence of *λ* and electronic DOS at Fermi level *N*(*E_f_*). At low doping (*x* = 0.05) where FS is a closed ellipsoid, the carrier concentration (*n*) is 3.5 × 10^20^ *cm*^−3^, slightly larger than the experimental one (~2.0 × 10^20^ *cm*^−3^)^13^. The corresponding *N*(*E_f_*) is about 2.64 states/Ryd/spin/(unit cell), and the calculated *λ* for this doping is even smaller (being 0.16). When increasing electron doping to *x* = 0.10, the *N*(*E_f_*) increased to 3.86 states/Ryd/spin/(unit cell), and the *λ* jumps to 0.26. Further increasing of doping will generally elevate *N*(*E_f_*) and *λ*, but the *λ* is still unable to exceed 0.30 at *x* = 0.14, although *n* at this doping level is already one order larger than the experimental one, which is probably unrealistic since the ambipolar doping nature of Cu tends to make electron concentration saturate at *x* = 0.1^30^. Even higher doping would in addition result in an complex Fermi surface structure which is probably inconsistent with experiments^19^,^39^.

Thus, given the experimental facts (with *n* ~ 2.0 × 10^20^ *cm*^−3^ and an ellipsoidal or cylindrical FS), the value of 0.28 corresponding to optimal doping has been the upper bound for *λ* and actually has overestimated it since the *n* is ~4 times larger than experimental one.

## Discussion

Our results are actually in line with recent experiments on epitaxial Cu*_x_*Bi_2_Se_3_ with thickness between 6 quintuple layers (QL) to 13 QL^29^, where superconducting transition is never observed down to 0.8 K even though the *n* is already comparable with bulk one (~10^20^ *cm*^−3^), suggesting that electron doping alone could not afford for the observed *T_c_* of ~4 K and in particular, a weak *e-ph* contribution to the observed superconductivity. Moreover, previous angle-resolved photoemission spectroscopy measurements of *e-ph* coupling in Bi_2_Se_3_ also suggest a relatively small *λ* of 0.25^47^ and 0.17^48^, which are very close to our first-principles results.

Therefore, we have to resort to other mechanisms besides *e-ph* coupling to recover the observed *T_c_* of ~4 K. Since electronic states of Cu*_x_*Bi_2_Se_3_ around Fermi level are dominated by *p* character^3^, we would expect minor contribution from spin-fluctuations either. On the other side, we note that the Cu-intercalated structure is formed both in bulk and films of Cu*_x_*Bi_2_Se_3_^29^,^31^, and the only difference is that disorder and inhomogeneity are strongly suppressed in latter case^29^. Indeed, there has been consistent observation of inhomogeneity of superconductivity in Cu*_x_*Bi_2_Se_3_^13^,^14^,^15^,^16^. And the superconducting shielding fraction reported also varies from group to group, in particular, Kriener *et al.*^40^ have shown that shielding fraction strongly depends on doping level *x* and *T_c_* exhibits an unusual monotonic decrease with *x*, raising the possibility of phase segregation.

Hence we could infer that the local inhomogeneity introduced by copper may play nontrivial role in the superconductivity of Cu*_x_*Bi_2_Se_3_^29^,^40^. Indeed, the intimate relationship between local inhomogeneity and superconductivity has been extensively studied in the context of high *T_c_* superconductors, and the possible enhancement of superconductivity by local inhomogeneities has been discussed recently by Martin I. *et al.*^49^ in the weak coupling BCS regime. Given many unusual properties of current system (relatively high *T_c_* compared to its low carrier density and the unexpected drop of shielding fraction for doping level *x* > 0.5)^40^, it is likely that the local inhomogeneity is indispensable to the superconductivity and may enhance *T_c_* of Cu*_x_*Bi_2_Se_3_.

Regarding the symmetry of the paring of superconducting Cu*_x_*Bi_2_Se_3_, no consensus has been reached since its first observation. Experimentally, point-contact spectroscopy measurements seem support the spin-triplet paring with odd-parity^15^, but results from scanning tunneling microscope and Andreev reflection spectroscopy show no evidence of characteristic zero-energy surface bound states^20^,^21^, suggesting the picture of s-wave paring. On the other hand, based on a 2 band model, Fu *et al.*^17^ argued that Cu*_x_*Bi_2_Se_3_ favors spin-triplet paring owing to its strong spin-orbit coupled band structure, and the possibility of phonon-mediated odd-parity paring has also been discussed theoretically^24^,^25^. Our first-principles results here suggest the paring of this system may be unconventional, and this unconventionality may come from the local inhomogeneity introduced by copper doping. This is because the superconducting transition hasn't observed in epitaxial Cu*_x_*Bi_2_Se_3_^29^, which would rule out the possibility that superconductivity is mediated by phonon modes derived from pure Bi_2_Se_3_.

In summary, we have presented a systematic study of phonons and role of *e-ph* coupling in the Cu doped Bi_2_Se_3_. Our results show that strongly renormalized zone center and zone boundary modes with electron doping would dominate the whole *e-ph* coupling process. Despite moderate *e-ph* coupling in this system, our robust first-principles calculations of *e-ph* properties for wide range of copper doping suggest that Cu*_x_*Bi_2_Se_3_ is not a conventional *e-ph* superconductor and *e-ph* coupling plays minor role in superconductivity of this system. We have also discussed the possible enhancement of *T_c_* by the local inhomogeneity introduced by copper doping. Above all, our results rule out a conventional phonon-mediated superconductivity in Cu*_x_*Bi_2_Se_3_ and point to a delicate interplay between *e-ph* coupling of parent compound Bi_2_Se_3_ and nontrivial role played by inhomogeneity.

## Methods

The *e-ph* properties have been obtained with the isotropic approximation to Migdal-Eliashberg theory^41^,^42^. In this framework, the phonon self-energy (Π**_q_**_*v*_) arising from *e-ph* coupling is expressed as^44^
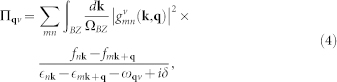
where 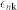 is the electronic energy with band index *n* and crystal momentum **k**, and *ω***_q_**_*v*_ is the vibrational frequency with branch index *v* and crystal momentum **q**. The *f_n_*_**k**_ is the Fermi-Dirac distributions, and *δ* is a positive infinitesimal. The 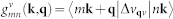 is the *e-ph* coupling vertex, where Δ*V***_q_**_*v*_ is the variation of the self-consistent potential induced by a collective ionic displacement. Note the spin degree has been incorporated into the band index.

The imaginary part of Π**_q_**_*v*_ corresponds directly to phonon half-width at half-maximum *γ***_q_**_*v*_, and the phonon mode-resolved *e-ph* coupling constant is given by
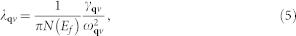
with *N*(*E_f_*) being the electronic density of states (DOS) at the Fermi level.

An accurate determination of phonon linewidths and hence *e-ph* coupling constant requires fine energy and momentum resolutions of electronic and phonon states as well as *e-ph* coupling matrix elements. We achieved this by the recently developed interpolation method through Maximally Localized Wannier Functions (MLWFs)^50^,^51^, which has been demonstrated to be extremely successful in addressing *e-ph* properties^52^,^53^,^54^,^55^ and other electronic properties where ultra high density of momentum is needed^56^,^57^. As first step of this method, we employs standard density functional theory (DFT)^58^ and density functional perturbation theory (DFPT)^59^ to obtain converged ground state electronic density and dynamical matrix. In this study, we use experimental crystal structure with *a* = 4.138 *Å* and *c* = 28.64 *Å*. Fully relativistic norm-conserving pseudopo-tentials is used for all the calculation here as relativistic corrections are necessary for a satisfactory quantitative description of topological insulator Bi_2_Se_3_. A kinetic energy cutoff of 35 Ryd with methfessel-paxton smearing widths of 0.01 Ryd and Monkhorst-Pack grids of 12 × 12 × 12 for k point sampling are used to ensure the convergence of total energy. The dynamical matrix is obtained on a relatively coarse grid of 4 × 4 × 4 phonon wave vectors (convergence has been achieved by comparing with phonon dispersion from 6 × 6 × 6 mesh).

Subsequently, the electronic and vibrational quantities obtained in first step are interpolated to dense grids which contains several tens of thousands of *inequivalent* phonon (electron) wave vectors. In this interpolation step, we first construct 30 spinor Wannier functions (using *p*-like atomic orbitals of Bi and Se) to span a subset of full Hilbert space around Fermi level, on which the operators in momentum space like Hamiltonian and electron-phonon coupling matrix are projected. After obtaining operators in Wannier representation, we then interpolate back to the momentum space (Bloch representation) to obtain the converged results^50^,^51^. By doing this way, the *e-ph* coupling constant *λ* is calculated on fine mesh grid containing 36 × 36 × 36 and 30 × 30 × 30 *inequivalent* electron and phonon momenta respectively. This dense grid ensures the convergence of *λ* to within 0.005.

## Author Contributions

X.L.Z. performed coding and calculations. X.L.Z., W.M.L. analyzed numerical results and contributed in completing the paper.
